# Lung Toxicity Analysis of Nano-Sized Kaolin and Bentonite: Missing Indications for a Common Grouping

**DOI:** 10.3390/nano10020204

**Published:** 2020-01-24

**Authors:** Martin Wiemann, Antje Vennemann, Wendel Wohlleben

**Affiliations:** 1 IBE R&D Institute for Lung Health gGmbH, 48149 Münster, Germany; vennemann@ibe-ms.de; 2 BASF SE, Advanced Materials Research, 67056 Ludwigshafen, Germany; wendel.wohlleben@basf.com

**Keywords:** nanotoxicology, aluminosilicate, alveolar macrophage, intratracheal instillation

## Abstract

Kaolin and bentonite (nanoclay NM-600) are nanostructured aluminosilicates that share a similar chemical composition, platelet-like morphology, and high binding capacity for biomolecules. To investigate if these material-based criteria allow for a common grouping, we prepared particle suspensions of kaolin and bentonite with a similar hydrodynamic diameter and administered them to NR8383 alveolar macrophages in vitro and also to a rat lung using quartz DQ12 as a reference material. Bentonite was far more bioactive in vitro, indicated by a lower threshold for the release of enzymes, tumor necrosis factor α, and H_2_O_2_. In addition, in the lung, the early effects of bentonite exceeded those of kaolin and even those of quartz, due to strongly increased numbers of inflammatory cells, and elevated concentrations of total protein and fibronectin within the bronchoalveolar lavage fluid. The pro-inflammatory effects of bentonite decreased over time, although assemblies of particle-laden alveolar macrophages (CD68 positive), numerous type-2 epithelial cells (immunopositive for pro-surfactant protein C), and hypertrophic lung epithelia persisted until day 21. At this point in time, kaolin-treated lungs were completely recovered, whereas quartz DQ12 had induced a progressive inflammation. We conclude that bentonite is far more bioactive than equally sized kaolin. This argues against a common grouping of aluminosilicates, previously suggested for different kaolin qualities.

## 1. Introduction

Kaolin (Al_2_Si_2_O_5_(OH)_4_) and bentonite (Al_2_H_2_Na_2_O_13_Si_4_) are platelet-like aluminosilicates (or mixed Si-Al-oxides), which are a challenging group of particles for risk assessment. Their chemical sum formula places them in the substance class of silicates, but their physical structure and biopersistence differ from pure silicates. Solubility studies on mineral fibers have shown that at acidic pH, mixed Si-Al-oxides have a significantly higher biodissolution rate and reduced biopersistence rate than pure Si-oxides [1]. In addition, the release of Al^3+^ ions may raise concerns, as recently considered by the European Chemicals Agency (ECHA) for the read-across of aluminum salts [2,3]. The toxicity assessment of aluminosilicates should, therefore, be based on a common evaluation of material properties, solubility data, and biological effects. Here, we investigate whether or not the high degree of structural and chemical similarity justifies an allocation of kaolin and bentonite to the same group of nanomaterials (NMs) according to current concepts also comprising in vitro and in vivo testing [4]. 

Kaolin is a natural aluminosilicate nanomaterial and may, therefore, be found at a low level in the ambient air. The material is used in megaton quantities for paper production but is also used by the chemical, pharmaceutical, and cosmetics industries for ceramic, rubber, plastic, and paint production [5]. Effects on kaolin mining workers have been studied in early case reports [6], and a low incidence of pneumoconiosis after prolonged exposure was reported [7,8,9]. Long-term workers exposed to 95% kaolin and low (<1%) quartz concentrations (mean lung burden of 63 mg/g tissue) exhibited a moderate interstitial fibrosis [10] such that kaolin appeared far less toxic than, e.g., quartz DQ12. Effects of kaolin on human lung were also suggested by animal experiments, although high doses were used in these studies: Sahu et al. [11] instilled 5 mg per mouse lung and detected “dirty white patches” and histological lesions but no signs of tumor formation. A mild fibrogenic effect of kaolin was reported by Rosmanith et al. (1989) and Wagner et al. (1986, 1987) [10,12,13] who also noted bronchoalveolar hyperplasia after inhalation. Under severe overload conditions and repeated instillation administration, kaolin with a particle size of 2 μm was found to induce a tumor formation [14]. Clearance from the lung of kaolin and other clay minerals mainly relies on physical mechanisms and also on solubilization [5].

Bentonite particles consist of highly colloidal and plastic clays mainly composed of montmorillonite and low, variable traces of quartz, cristobalite, and feldspar. Similar to kaolin, bentonite comes in flat, platelet-like particles. The basal molecular structure of bentonite comprises two (tetrahedral) silica sheets that enclose an (octahedral) alumina sheet in between [5]. The material is industrially used as a binding foundry sand in molds, as grease, or as an absorbing material in wastewater. As part of many consumer products, it is also incorporated in ceramics, and serves as a stabilizer or extender in adhesives, paints, cosmetics, and medicines [5]. Bentonite forms thixotropic gels with water and is used as a clarifying agent in wine making [15] or vegetable oil as a bonding agent. Considering its widespread use, the exposure to bentonite of the general population is low. However, the occupational exposure to bentonite mining dust may be higher, and several studies have investigated the effects of bentonite on the lung. Bentonite and montmorillonite (with low quartz content) caused a transient local inflammation, edema, and increased lung weight [16]. 

According to their similar chemical structure, and their platelet-like morphology with opposite charges of lateral and edge surfaces, kaolin and bentonite may induce similar biologic effects inside the lung. However, a direct comparison of the bioactivity of kaolin and bentonite nanomaterials has not yet been carried out for similarly sized materials at the nano level. Of note, the particle size-distribution is of pivotal importance for both in vivo and in vitro testing, as it determines lung deposition, biodistribution or, in terms of deposition and gravitational settling, the availability to interact with cells. Here, we compare the effects of kaolin and bentonite suspensions with mean particle sizes being highly similar. The bentonite quality used for this study is provided by Joint Research Center (JRC) as a characterized standard nanomaterial (“nanoclay NM-600”) [17] but, to the best of our knowledge, has not yet been investigated for its biological properties. A rat alveolar macrophage assay was used to describe in vitro effects. A single concentration was then administered to the rat lung to analyze bronchoalveolar lavage fluid (BALF), lung histology, and particle distribution in lung tissue.

## 2. Materials and Methods 

### 2.1. Particle Characterization and Preparation of Suspensions 

Bentonite (NM-600 Nanoclay, CAS no. 1302-78-9) is a hydrated sodium calcium aluminum magnesium silicate hydroxide with a quartz content of up to 3%. The material was provided by the Joint Research Center (JRC, Ispra, Italy), characterized by JRC [18] and also in a previous publication [4]. In brief, bentonite as a powder material has a mean particle size of 288 nm (percentile d10: 631 nm, percentile d50: 166 nm, percentile d90: Mean 87 nm). The specific surface size (BET) amounted to 51.9 m^2^/g. A quartz content of up to 3% was supposed. Energy dispersive X-ray spectroscopy (EDX) results showed that a thin layer of the NM-600 bentonite contains (in mass %): Ca (0.29), Fe (1.61), Na (2.85), Mg (1.29), Al (8.61), Si (22.25), O (62.84), and S (0.27). 

Kaolin (CAS no. 1332-58-7) was provided and characterized by the NanoGRAVUR consortium [4]. A highly similar material (JRC-IRMM385), provided by the NanoDefine consortium (not used here) was characterized by Babick et al. [19]. The specific surface size of this type of kaolin (BET) was 24 m^2^/g. Quartz DQ12 and corundum particles had been characterized before and were suspended as described [20]. Kaolin and bentonite (2.4 mg/mL) were suspended in distilled pyrogen-free H_2_O (dH_2_O) by vortexing, followed by dispersion with ultrasonic energy for 10 s and 5 x 1 min on ice (300 s), respectively. Therefore, a VibraCell™ (Sonics & Materials, Danbury, CT, USA) was operated at 50 W. The hydrodynamic diameters of all particle suspensions were measured by particle tracking analysis in dH_2_O and also in cell culture media (see below) using a NanoSight LM10 instrument equipped with a blue laser (405 nm), an Andor CCD camera, and NanoSight software (NTA 3.1, Malvern Instruments GmbH, Herrenberg, Germany). The z-potential measurements were carried out with a Zetasizer Nano ZS (Malvern Instruments GmbH, Herrenberg, Germany).

### 2.2. In Vitro Toxicity Test

The rat alveolar macrophage cell line NR8383 was cultured at 37 °C and 5% CO_2_ in 175 cm^2^ culture flasks in F-12K medium prepared from powder medium (Sigma-Aldrich, N3520, Germany), supplemented with 15% heat-inactivated fetal calf serum, glutamine (2 mM), and 100 U penicillin and 10 mg/mL streptomycin. Experiments were performed as described with minor modifications [21]. In brief, cells were incubated with increasing concentrations of particles in serum-free F-12K medium. Therefore, the aqueous particle stock suspensions were diluted to a working concentration of 360 μg/mL. These aqueous suspensions were further diluted in an equal volume of double-concentrated F-12K cell culture medium or double-concentrated Krebs–Ringer buffer containing 2 mmol/L glucose (KRPG), to achieve cell compatibility of the suspensions. F-12K medium or KRPG were used for all further dilution steps. NR8383 cells (3 × 10^5^ per well of a 96-well plate) were incubated with increasing concentrations of particle suspensions prepared in serum-free F-12K medium for 16 h. Supernatants were retrieved and centrifuged at 200× *g* for 10 min. Each supernatant was analyzed for lactate dehydrogenase activity (LDH), glucuronidase activity (GLU), and tumor necrosis factor α (TNFα) using a specific enzyme-linked immunosorbent assay (ELISA) for rat TNF-α (bio-techne, Wiesbaden, Germany). LDH was measured with the Cytotoxicity Detection Kit (Merck KGaA, Darmstadt, Germany) and GLU was measured photometrically using *p*-nitrophenyl-β-D-glucuronide as a substrate. Both enzyme activities were expressed as a % of the positive control value, which was obtained by adding 0.1% triton X-100 to an equal number of non-particle-treated cells. Bioactive TNF-α was measured indirectly via induction of apoptosis/necrosis in L-929 fibroblasts in the presence of actinomycin D and was expressed as % killing activity [22]. The release of H_2_O_2_ was measured 90 min after the addition of particles, which were suspended in KRPG. H_2_O_2_ concentration was determined quantitatively using resorufin as a detection reagent in the presence of horseradish peroxidase. All assays were run in 96-well plates and repeated three times. Vehicle-treated cells were used as negative controls. Cell-free wells were processed in the same way and used to exclude particle interference.

### 2.3. Animal Experiments

The animal experiments were conducted at the animal facility of the University Clinics of Essen, Germany and ethically approved by LANUV (Dortmund, Germany, Accession No. 84-02.04.2022.A157). Female Wistar rats, strain WU, weighing 200–250 g (Charles River Laboratories, Sulzfeld, Germany), were maintained in a 12 h lights-on lights-off environment. Food and water were provided ad libitum. Particles used for intratracheal instillation experiments were suspended as described for in vitro experiments in dH_2_O at a concentration of 2.4 mg/mL. All instillation fluids were prepared on the day of the experiment. The animals (*n* = 5 per group) were anaesthetized with isoflurane and received 500 μL of instillation fluid (containing 1.2 mg (*w*/*v*) of either kaolin, bentonite, or quartz DQ12) intratracheally under visual control. After three and 21 days, the animals were deeply anaesthetized with a mixture of ketamine and xylazine. Citrate blood (1 mL) was retrieved from the left ventricle, and blood cell analysis was carried out with a Sysmex KX21N (Sysmex GmbH, Hamburg, Germany). The animals were then bled from the *Aorta descendens* and BALF was prepared from the right lung by repeated washing with 0.9% NaCl (5 × 3 mL). Thereafter, the left lung was filled with 3 mL cryomatrix (Thermo Shandon Ltd., Runcorn, UK), excised, and snap-frozen in liquid nitrogen.

BALF was centrifuged (200 ×g, 4 °C, 10 min), cell pellets were re-suspended, and cells were counted with a Coulter counter (model Z2, Beckman Coulter GmbH, Krefeld, Germany). The cell viability was determined by trypan blue testing. Differential cell counts of at least 400 cells per animal were obtained after May-Grünwald and Giemsa staining of cytospin preparations, as described [23]. The total BALF protein was measured with the Lowry method, using bovine serum albumin as a standard. Fibronectin was detected by a specific ELISA [24]. 

### 2.4. Immunocytochemistry and Microscopy

Cryo-sections (7 μm) were cut from the left lung, dried onto glass slides, and stored at −20 °C. Frozen sections were post-fixed with 3.7% phosphate-buffered formaldehyde for 10 min. Sections were rinsed in phosphate-buffered saline (PBS), followed by quenching of endogenous peroxidase with 1% H_2_O_2_ in PBS for 10 min and treated with 3% bovine serum albumin (fraction V) for 1 h at room temperature. An anti-CD68 antibody (AbDSerotec, diluted 1:100 in PBS, 1% BSA) and an anti-pro-surfactant protein C antibody (antibodies-online, diluted 1:400 in PBS, 1% BSA) was used to label alveolar macrophages and type-2 epithelial cells, respectively. Rabbit anti-nitrotyrosin antibody (Merck Millipore No.: 06284, diluted 1:200) was used to label nitrosylated proteins. Bound specific antibodies were labelled with biotinylated anti-mouse IgG (Jackson Immuno Research Labs, West Grove, PA, USA) to detect CD68, and biotinylated anti-rabbit IgG (Vector Laboratories, Burlingame, CA, USA) to detect surfactant protein C and nitrotyrosine. Avidin-biotin horse radish peroxidase-based detection systems (Vector Laboratories, No.: PK-4001) followed by substrate staining (Vector blue for CD68 and nitrotyrosine, Vector laboratories, No.: SK-4700) and AEC for surfactant protein C (Vector Laboratories, No.: SK-4205) were used. Nitrosylated tissue was prepared as a positive control by bathing sections in a mixture of 1 mM NaNO_2_/1 mM H_2_O_2_ in 100 mM sodium acetate, pH 5. All sections were finally rinsed in phosphate buffered saline (PBS) and cover slipped with aqueous mounting medium (Immu-Mount, Fisher Scientific GmbH, Schwerte, Germany). Micrographs were taken with a Retiga 2000R camera and Q capture imaging software (Q Imaging Corporation, Surrey, BC, Canada) mounted on an Olympus BX51 microscope (Olympus Germany GmbH, Hamburg, Germany). Unstained and immuno-stained sections were also viewed with enhanced dark field microscopy (DFM) using an appropriate DFM condenser (CytoViva Inc., Auburn, AL, USA). Polarization microscopy was carried out with two perpendicularly oriented linear polarizing filters, which were inserted into the optical path of the microscope.

### 2.5. Statistics

At least three independent repetitions of in vitro experiments were carried out. Results for each concentration were compared to non-particle treated controls by two-way analysis of variance (ANOVA) and Dunnets’s multiple comparisons test, using GraphPad Prism 6.01. Particle size data were compared using an unpaired t-test. In vivo results are based upon five animals per group; all data are expressed as mean ± standard deviation (SD). BALF data were compared pair-wise to the vehicle control group by one-way ANOVA and post-hoc Dunnett’s multiple comparison test. For all experiments, *p* ≤ 0.05 was considered significant.

## 3. Results

### 3.1. Particle Size after Ultrasonic Dispersion 

**Figure 1:**
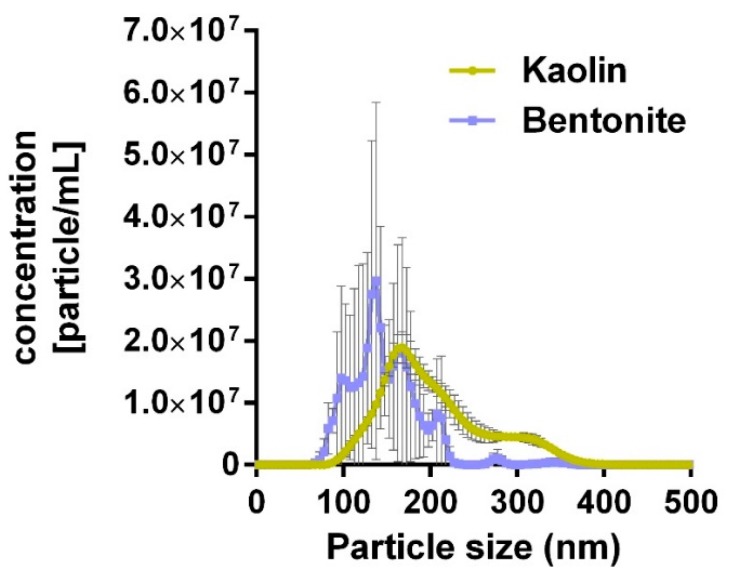
Particle size distribution of kaolin and bentonite particles dispersed in distilled H_2_O as analyzed with optical particle tracking. Kaolin and bentonite were dispersed for 10 and 300 s, respectively (see Method section for details).

Aqueous kaolin and bentonite suspensions were subjected to differential ultrasonic treatment (kaolin: 10 s; bentonite: 300 s). This led to similar, non-significantly different mode values, ranging from 100 to 350 nm (Figure 1). Table 1 shows that the transfer from H_2_O to KRPG elicited a slight agglomeration, with the mode values of kaolin and bentonite suspensions remaining similar and not significantly different. The transfer of the aqueous dispersions into the F-12K medium led to a more pronounced agglomeration of both aluminosilicates. While the bentonite agglomerate reached hydrodynamic diameters of 350 nm (Table 1), kaolin agglomerates were larger and no longer measurable with optical particle tracking; agglomerates were, however, partly visible with phase contrast optics (see below). A de-agglomeration of kaolin could be achieved by an additional 10 s lasting ultrasonication, and these values are shown in Table 1 for comparison. In line with these observations, the z-potential of bentonite and kaolin was strongly negative in H_2_O and KRPG, and became less negative in the F-12K medium (Table 1).

### 3.2. In Vitro Study

**Figure 2:**
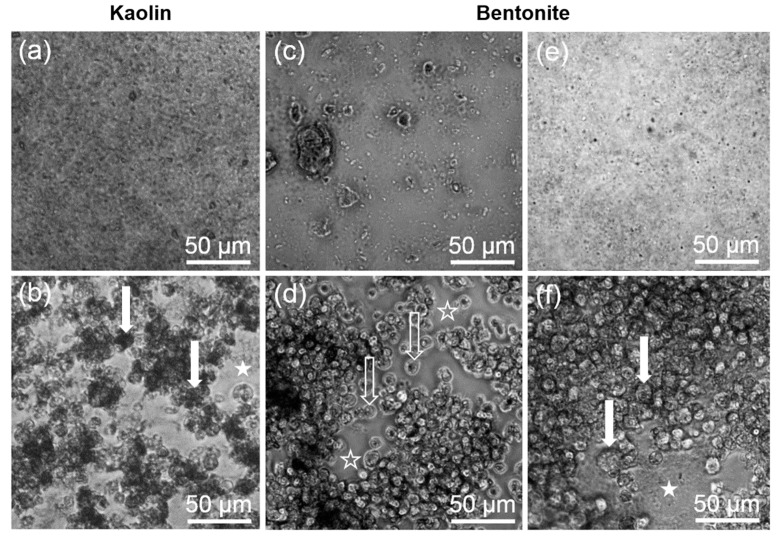
Gravitationally settled kaolin and bentonite particles, and uptake by NR8383 cells after 16 h. (**a**,**c**,**e**) Settled particles in cell-free control wells. (**b**,**d**,**f**) NR8383 cells incubated for 16 h with the same particles as shown above. (a,b) Kaolin (180 µg/mL) after ultrasonic dispersion for 10 s. (c,d) Bentonite (180 µg/mL) after ultrasonic dispersion for 10 s. (e,f) Bentonite (180 µg/mL) after ultrasonic dispersion for 300 s. Filled arrows point to dark, particle-filled cells (b), or deteriorated cells with granular appearance (f). Filled asterisks mark areas where settled, non-ingested particle agglomerates are visible (b,f). Open arrows (d) show largely uncompromised cells; open asterisks (d) mark areas that had been cleared from particulate matter by phagocytosing cells.

The in vitro toxicity of kaolin and bentonite was measured with the alveolar macrophage assay in comparison to corundum and quartz DQ12. Under cell-free conditions, corundum and quartz DQ12 appeared as microscopically visible particles at the bottom of the culture vessel due to gravitational settling (not shown). Precipitates of kaolin and bentonite particles and/or agglomerates were visible as well (Figure 2). Of note, the morphology of both aluminosilicates differentially dispersed by ultrasonic treatment (see above) appeared highly similar. By contrast, a brief 10 s lasting ultrasonic treatment of bentonite led to numerous large bentonite particles that could not be phagocytized by the cells (Figure 2c) and whose effects were not further investigated. Both aluminosilicates, as well as corundum and quartz DQ12 particles (not shown), were largely engulfed by the NR8383 cells during the 16 h lasting incubation period (Figure 2b,f), and at least high concentrations of kaolin and bentonite led to visible signs of cytotoxicity such as low contrast and/or a granular appearance of the cells (Figure 2b,f).

**Figure 3:**
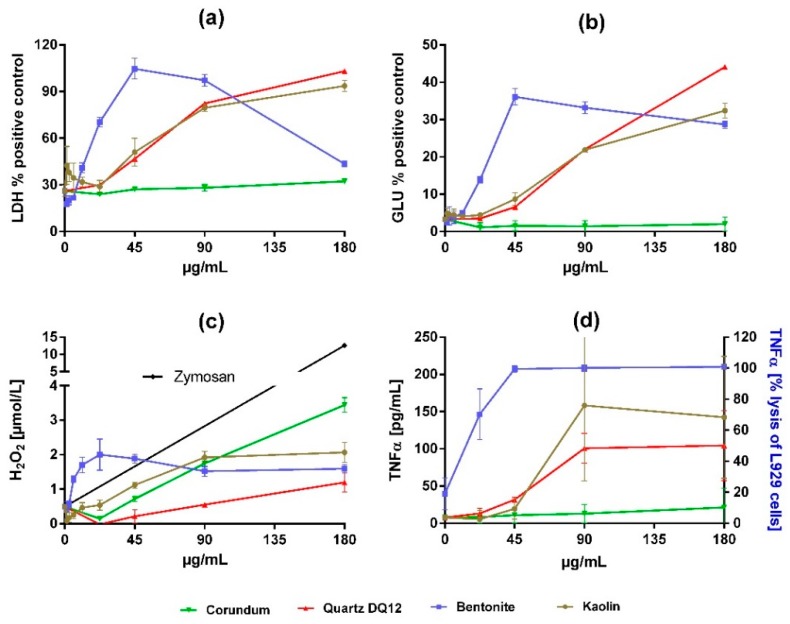
Dose-dependent effects of corundum, quartz DQ12, bentonite, and kaolin on NR8383 alveolar macrophages in vitro. Cultures of 3 × 10^5^ cells per well were incubated with particle concentrations, as indicated by the graphs. After 16 h, culture supernatants were assessed for (**a**) activity of lactate dehydrogenase (LDH) in percent of triton X-100 lysed control cells, (**b**) activity of glucuronidase (GLU) in percent of triton X-100 lysed control cells, (**c**) extracellular H_2_O_2_, and (**d**) tumor necrosis factor α (TNFα), measured by a specific enzyme-linked immunosorbent assay or, in the case of bentonite, by the lysis of L929 fibroblasts. The black line in (c) shows H_2_O_2_ release upon 180 µg/mL zymosan (positive control). Values are means ± SD from three independent experiments.

In vitro effects of all particles are shown in Figure 3 and Table 2. Corundum particles elicited nearly no cytotoxicity but induced a moderate dose-dependent formation of H_2_O_2_. By contrast, quartz DQ12 was strongly cytotoxic as indicated by the release of LDH and GLU. The pro-inflammatory effect of quartz DQ12 was reflected by a strong induction of TNFα, although the formation of H_2_O_2_ was typically low. The findings for corundum and quartz DQ12 are in accord with historical records.

Kaolin elicited dose-dependent effects on NR8383 alveolar macrophages in vitro, which were highly similar to those of quartz DQ12 with respect to the release of LDH, GLU, and TNFα, while H_2_O_2_ formation was increased (Figure 3, Table 2). 

Effects of bentonite were more pronounced. The dose−response curves for the release into the medium of LDH, GLU, TNFα, and H_2_O_2_ were much steeper and shifted leftward. Interestingly, we found clear maximum values for the activities of LDH and GLU at a concentration of 45 µg/mL. Based on the significant low adverse effect concentration measured for LDH (Table 2), bentonite was 4-times more cytotoxic than kaolin.

### 3.3. In Vivo Study

**Figure 4:**
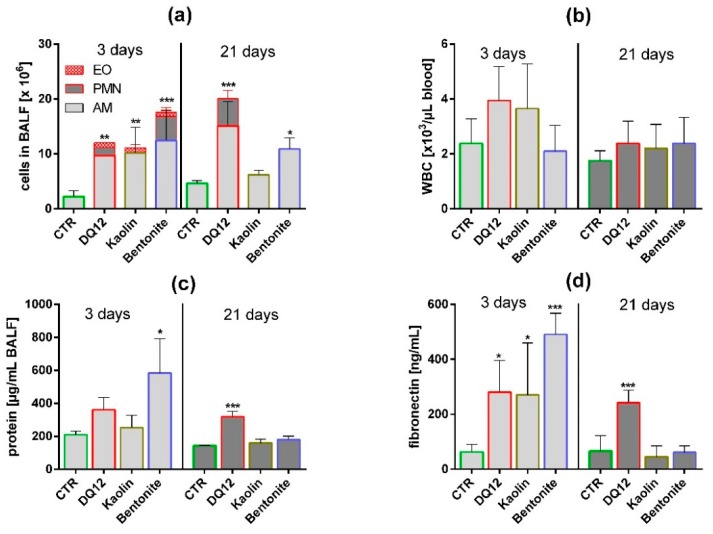
In vivo effects of kaolin, bentonite, and quartz DQ12 on lung and peripheral blood. Analysis of the bronchoalveolar lavage fluid (BALF) and of white blood cells (WBC) in peripheral blood (5 animals per group) 3 and 21 days after intratracheal instillation of 1.2 mg (all particles). The vehicle control (CTR) received 0.5 mL H_2_O only. (**a**) Differential cell counts revealed changes in the numbers of eosinophils (EO), neutrophilic granulocytes (PMN), and alveolar macrophages (AM). (**b**) Number of WBC in citrate blood. (**c**) Concentration of total protein in BALF. (**d**) Concentration of fibronectin in BALF. *: *p* < 0.05, **: *p* < 0.01, ***: *p* < 0.001.

**Figure 5:**
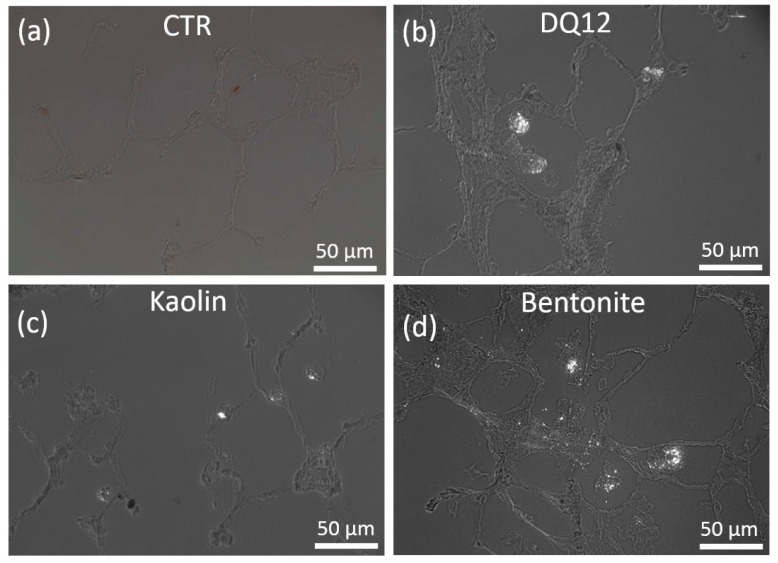
Detection of polarizing particles in quartz DQ12, kaolin, and bentonite-laden rat lungs. Cryo-sections investigated by polarization microscopy (polarizers 90° crossed). Animals were intratracheally instilled with vehicle (**a**, CTR), or 1.2 mg of either quartz DQ12 (**b**), kaolin (**c**), or bentonite (**d**), and were sacrificed after 21 days. Bright particulate matter occurred in discrete macrophage-like cells facing the alveolar lumen. In bentonite-treated lungs (**d**), particles were less concentrated and occurred with alveolar septa.

**Figure 6:**
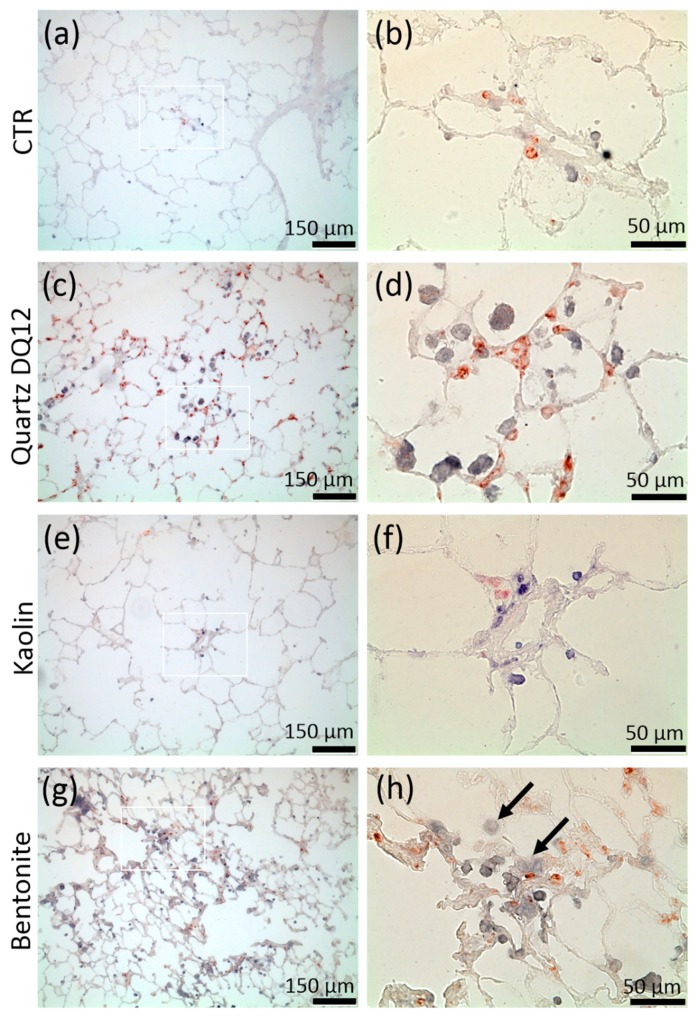
Distribution of alveolar macrophages (AM) and pro-surfactant protein C positive type-2 epithelial cells (AEC-II) in rat lungs treated with kaolin, bentonite, or quartz DQ12. Animals were intratracheally instilled with vehicle (CTR; **a**,**b**), or 1.2 mg of either quartz DQ12 (**c**,**d**), kaolin (**e**,**f**), or bentonite (**g**,**h**) and sacrificed after 21 days (a,h). Alveolar macrophages (AM) and AEC-II were indirectly stained with antibodies against CD68 (blue) and anti-pro-surfactant protein c antibodies (red). Figure parts b, d, f, and h represent the white boxes in a, c, e, and g. Assemblies of AM and increased expression of pro-surfactant protein c are evident in quartz DQ12-treated (c,d) and bentonite-treated lungs (g,h). Hypertrophic epithelia are seen in bentonite-treated (g), but not in quartz DQ12-treated, lungs.

A single dose of 1.2 mg per rat lung was chosen to compare the effects of bentonite, kaolin, and quartz DQ12 in vivo. This dose had been successfully used to obtain significant changes in previous experiments with quartz DQ12 as a positive control [23]. The same dose may also be estimated from the most effective concentration of kaolin in vitro (180 µg/mL, i.e., 36 µg/well), which calculates to a mean cellular dose of 120 pg/cell (36 µg divided by 3 × 10^5^ cells per well). If multiplied by 10^7^, which is a typical number of alveolar macrophages in the non-compromised rat lung [25], a dose of 1.2 mg is obtained. Considering the even higher bio-activity of bentonite in vitro, we expected significant changes in BALF and, at least in part, also in the lung histology. Main results are shown in Figure 4, Figure 5 and Figure 6 and values are shown in Tables S1–S3.

#### 3.3.1. Quartz DQ12

Quartz DQ12 was used as a positive control and elicited typical increases in the numbers of lavagable alveolar macrophages (AM), and neutrophilic granulocytes (PMN) relative to vehicle control (Figure 4a). Effects were significantly different from the control on day 3 and became further augmented on day 21, especially with respect to increased numbers of neutrophilic granulocyte (PMN), indicating the typical quartz-driven progressive inflammation. Low amounts of eosinophils (EO) on day 3 were accompanied by non-significant changes in white blood cell numbers in 2/5 animals (Figure 4b). Total protein (Figure 4c) and fibronectin concentrations (Figure 4d) were also significantly elevated. Quartz DQ12 particles occurred mainly in alveolar macrophages (Figure 5b), and the histological examination of hematoxylin eosin (HE)-stained tissue confirmed that quartz DQ12-treated lungs, in contrast to the control lungs, exhibited pronounced assemblies of enlarged or partially deteriorated CD68-positive AM within the lung parenchyma (Figure 6d). DQ12 particles in the lung were hardly detectable by brightfield or enhanced darkfield microscopy (Figure S1). However, typical birefringent particles not seen in control animals appeared in AM under polarized light (Figure 5). Quartz DQ12 treatment also increased the number and staining intensity of pro-surfactant protein C (pSP-C) positive type-2 epithelial cells (Figure 6). The pSP-C positive cells occurred throughout the lung parenchyma with no spatial relation to regions crowded by AM. Overall, the changes upon 1.2 mg quartz DQ12 were deemed typical for the early response of the rat lung for this fibrogenic type of quartz.

#### 3.3.2. Kaolin

Kaolin induced an increase in AM and EO counts in BALF, highly similar to quartz DQ12, but, in contrast to the latter, had nearly no effect on PMN counts (Figure 4, Table S1). The fibronectin and total protein concentrations were elevated in BALF on day 3; all effects had fully recovered on day 21. At this point, in time kaolin was localized in AM (Figure 5), and no structural changes in the lung parenchyma (Figure S1) and in the numbers of CD68-positive AM or pSP-C positive cells were noted (Figure 6). Overall, 1.2 mg kaolin induced a transient macrophage-based hypercellularity in the rat lung, but, unlike quartz DQ12, elicited neither a (progressive) inflammation nor a structural change of the lung parenchyma.

#### 3.3.3. Bentonite

In contrast to kaolin, bentonite had a very strong effect on the rat lung on day 3. Even compared to quartz DQ12 positive control, AM and PMN counts were 1.15- and 3.1-fold higher, respectively. In addition, the increases in total protein and fibronectin in BALF outscored the values of quartz DQ12 by far (Figure 4), indicating lung cell damage and/or epithelial leakage. Lung biopsy revealed numerous opaque and even petechia-like sites at the outer lung surface (Figure S2) on day 3. Interestingly, these macroscopic signs of damage and also most other inflammatory markers had decayed down to the control level on day 21, except for the still increased AM counts, which were confirmed by histology on days 3 and 21 (Figure S1). In contrast to quartz DQ12-treated lungs, alveolar septae in bentonite-laden lungs appeared hypertrophic with beginning hyperplasia. Numerous pSP-C-positive type-2 cells were found close to assemblies of CD68 positive AM (Figure 6). Interestingly, the CD68 staining intensity of many alveolar macrophages was low in bentonite-laden lungs. Weakly CD68-positive cells with bubble-like inclusions were also found within alveolar septae and appeared equivalent to similar formations seen in HE-stained tissue (Figure S1, Figure 6). By means of polarization microscopy, but hardly by DFM (Figure S1), birefringent particles were seen in alveolar macrophages. Unlike quartz DQ12 or kaolin treatment, bentonite particles also appeared within alveolar septae (Figure 5). Therefore, a staining for immunoreactive nitrotyrosin was carried out, showing immunopositive areas in alveolar septae not seen in controls (Figure S4). Overall, bentonite elicited a very strong, though transient, inflammation of the lung along with beginning structural changes of the lung epithelium. 

## 4. Discussion

In this paper, we produced aqueous suspensions from kaolin and bentonite powders with a highly similar particle size distribution down to the nanosize, to better compare the bioactivity of both aluminosilicates. Under these conditions, bentonite was about fourfold more bio-active than kaolin in the alveolar macrophage assay with respect to cytotoxicity and TNFα induction. In addition, in the rat lung, bentonite was far more bio-active as it elicited a pronounced, though transient, inflammation, which outscored the early effects of quartz DQ12 on day 3 but, in contrast to the latter, was not progressively inflammogenic. The effects of kaolin were moderate, transient, and resembled a typical foreign body reaction.

In principle, these findings are in line with earlier studies, which were, however, not specially designed to compare nano fractions of similarly sized particles. In macrophage-like P388D_1_ cells, bentonite in the presence of 4% serum was more cytotoxic than kaolin [26]. In addition, RAW267.4 cells in the presence of 10% serum were lysed by ≥25 µg/mL bentonite but were resistant against 100 µg/mL kaolin [27]. Considering the serum-mediated attenuation of silica toxicity, these findings are in line with our findings on NR8383 macrophages, which were lysed upon 11.25–22.5 µg/mL bentonite and 45–90 µg/mL kaolin, respectively. Bentonite (100 µg/mL) was also readily cytotoxic for primary neurons under serum-free conditions, but not for cultured NIE-115 cells in the presence of 10% serum [28]. Bowman and co-workers suggested that the toxicity of bentonite requires a direct contact of particles and cells because toxic effects were not observed when cells and particles were separated by a membrane [27]. Overall, bentonite is more cytotoxic than kaolin for NR8383 alveolar macrophages and many other cells. 

The comparatively strong effect of kaolin on NR8383 cells found here differs from earlier findings, which demonstrated a cytotoxicity of 100 µg/mL kaolin on NR8383 cells after 5 days only [29]. Again, the low cytotoxicity of kaolin in that study may be explained by the presence of 10% serum, known to mitigate the in vitro response of silica [30,31]. However, the attenuated cytotoxicity described by Gao and co-workers [29] is in line with the moderate in vivo response seen here in the rat lung for kaolin, suggesting that the milieu of the inner lung surface mainly composed of lung surfactant and lung lining fluid mitigates kaolin effects. Especially, dipalmitoylphosphatidylcholine (DPPC), the major phospholipid of the lung surfactant, inhibited cytotoxic effects of quartz and kaolin [29]. The lower toxicity of kaolin compared to quartz, as found here in vivo, has also been documented in cell studies [26]. However, bentonite particles bind to proteins and surfactant components as well [32], but for bentonite, a protein and/or lipid corona formation was not sufficient to inhibit inflammatory effects. Interestingly, the CD68 staining intensity of many alveolar macrophages was low in bentonite-laden lungs (c.f. Figure 6h), suggesting beginning cell damage upon bentonite inclusion. Meanwhile, there is compelling evidence that the toxicity of silica material, including quartz and amorphous silica as well, is linked up with the steric organization of superficially located silanol groups responsible for membranolytic activity, cell surface receptor interaction, and proteasome activation [31]. As kaolin and bentonite share tetrahedric SiO_2_ layers at their outer surface [33], a silica-like bioactivity may be expected for both aluminosilicates, whereby bentonite with its larger BET surface (51.9 vs. 24 m^2^/g) should be about twice as reactive. Furthermore, a bolus administration of particles with a large surface may adsorb high amounts of lung surfactant compromising normal lung function [34]. Such a disturbance of the inner lung milieu may especially apply for bentonite, which adsorbs proteins faster and more effectively than kaolin (Haase, personal communication). Adsorption of proteins by bentonite may also explain the biphasic responses observed here for the activities of LDH and GLU in cell culture supernatant. It may be speculated that these enzymes are bound and inactivated by bentonite and that this effect is especially evident under serum-free conditions.

The direct comparison of in vitro and in vivo results is always impeded by different observation periods (16 h in vitro versus 3 or even 21 days in vivo), different dose rates (kaolin agglomerated in F-12K medium and settled completely), and by the fact that the lung is a dynamic and open system into which cell populations such as monocytes and PMN may invade from the blood stream. Nevertheless, the pronounced cytotoxic effects found for bentonite in vitro seem to be reflected by the elevated concentrations of total protein (2.8-fold of control) and of fibronectin in BALF (6.8-fold of control) on day 3. These proteins may originate from the damaged or leaky lung epithelia, as suggested by histology (see Figure 6g), but also from deteriorated macrophages, which, in a compromised lung, are known to contain fibronectin [35]. Quartz DQ12 elicited less pronounced increases in total protein and fibronectin (1.7- and 3.5-fold of control, respectively) and this difference appears in accordance with its lower bio-activity in vitro. In addition, the pro-inflammatory effect of bentonite indicated by high PMN counts was 3.1-fold larger than the quartz DQ12-induced recruitment of PMN on day 3, possibly reflecting the discrepancy in TNFα expression in vitro, although PMN invasion appears to involve several mediators [36]. Interestingly, the in vitro assays partly overestimated the effects of kaolin in the lung, especially when compared to the effects of quartz DQ12: While all in vitro dose–response curves of quartz DQ12 and kaolin were largely congruent, in vivo only elevated AM counts, and the concentration of fibronectin was nearly identical, whereas PMN counts and the total protein concentration were far lower and hardly different from the vehicle control. Considering the histological pictures of the lung (Figure 5c and Figure 6e), it appears that kaolin does not compromise the lung epithelium known to be involved in the recruitment of PMN from the blood [37]. Overall, the in vitro in vivo comparison shows that the alveolar macrophage assay is a valuable tool to predict early effects of (nano)particles on the alveolar macrophage population but has its limitations if complex biological processes are to be predicted.

There were also some differences between kaolin and bentonite concerning particle localization in lung tissue. The detection of small silica particles in lung tissue even by enhanced darkfield microscopy was cumbersome due to the high transparency of silica (Figure S1). However, crystalline quartz alters the plane of polarized light and is, therefore, detectable by polarization microscopy. Here, also kaolin and bentonite particles were visualized as bright objects under the polarization microscope, possibly because their layer structure changes the plane of the polarized light. By this approach, quartz DQ12, kaolin, and bentonite particles were detected in macrophage-like cells. Only bentonite particles were found also within alveolar septae, suggesting their uptake into epithelial cells or interstitial macrophages. This finding is in line with an earlier study on the subcellular localization of bentonite (sized 0–2 µm) in the rat lung [38], showing that epithelial cells within so-called “storage foci” contained numerous stacked, lamellar particles identified as bentonite by energy-dispersive X-ray analysis. Interestingly, many of these inclusions were not surrounded by membranes and were located in close contact to unrestrained cytoplasmic organelles and cytosolic components. Of note, this situation was obtained three or six months post-administration of bentonite. Given the acute cytotoxicity and acute lung toxicity of this aluminosilicate, and also the unexpected recovery from effects in the lung as seen here, we hypothesize that bentonite, by contacting cells and/or tissue fluids, transforms into a biocompatible material over time. The biocompatible stage may require an equilibrium to be reached for water adsorption, ion exchange processes, and protein and/or lipid corona formation. Further equilibration experiments followed by macrophage testing is required to better understand bentonite’s mode of action.

Aside from differences based on surface size, surfactant binding, and protein adsorption, the layered structure of montmorillonite, the major constituent of bentonite, differs from that of kaolinite as it bears the gippsite layer between two silica sheets. This feature enables the material to adsorb high amounts of H_2_O, leading to a so-called “expanding lattice” with a variable thickness of 0.96 to 2.14 nm [33]. While this process may disturb the water balance and osmolarity of ingesting cells or tissues exposed to dry bentonite, it may be less relevant in our experiments with well-dispersed, H_2_O-equilibrated materials. However, the special arrangement of the gippsite layer structure of montmorillonite also allows for a considerable ion exchange capacity [33], which amounts to 184 mEq for bentonite but only to 4 mEq for kaolin [27]. Ion exchange of bentonite seems to be driven by Si and Al ions leaving the lattice and being replaced by Ca^2+^, Na^+^, and Mg^2+^ ions entering the bentonite nanostructure. It is tempting to speculate that these changes take place at least in part within alveolar macrophages and/or epithelial cells (c.f. Figure 5) and that they contribute to ionic instability and eventually to cytotoxicity seen in NR8383 cells. In addition, tissue hypertrophy and/or swelling in the vicinity of bentonite-triggered alveolar macrophage assemblies may be caused by local osmotic effects emanating from bentonite particles (c.f. Figure 6g). Local ionic imbalances around bentonite particles may therefore be of importance for the toxic potential of this aluminosilicate and, especially, an unbalanced retrieval of Ca^2+^ and/or Mg^2+^ ions may destabilize a cell. As larger differences in the z-potential of kaolin and bentonite were not found (Table 1), this parameter appears of minor importance. Further studies are in progress to identify the transformation of aluminosilicates in situ.

Interestingly, an unproportional release of Al and Si ions has been demonstrated from kaolin and bentonite flushed by a pH 4.5 phagolysosomal simulation fluid (Keller et al., this special issue), with Si ions being released faster than Al ions. In comparison to kaolin, the bentonite dissolution rate of 33 ng/cm²/h (calculated dissolution halftime: 1.7 d) was much faster than the dissolution rate of kaolin (9.8 ng/cm²/h, calculated dissolution halftime: 12.3 d), and both were categorized as “partially dissolving,” such that the toxicity of both ions and particles needs to be considered. Al ions are known to act geno- and neurotoxic [39] and their local release from aluminosilicates, which is about five times faster from bentonite compared to kaolin (Keller et al., this special issue), may contribute to cytotoxic effects in the lung as well. Although the administration of 46.6 µg Al_3_(SO_4_)_2_ to the rat lung had no adverse effect on BALF parameters [40], and even 500–1000 µM Al^3+^ were needed to observe a growth retarding effect on smooth muscle cells [41], a contribution of Al ions to bentonite toxicity cannot completely be ruled out, if local concentrations reach high values. 

A further effect of bentonite and kaolin may be based on oxidative damage, and this may be attributed to the edges of the aluminosilicate lattice. Of note, a platelet-like kaolin fostered the generation of radical oxidative species and genotoxic events in vitro, whereas a more spherical kaolin had lower effects [42]. Here, the bentonite-treated NR8383 cells produced more H_2_O_2_ than kaolin-treated cells, which is in line with an earlier study measuring particle-induced chemiluminescence [43]. In another study [4], we employed the ferric reduction ability of serum (FRAS) assay and two electron paramagnetic resonance (EPR) assays, and found that the oxidative power of bentonite outscored that of kaolin and several other silica nanoparticles about 4–8 fold. On the other hand, the carbonylation of cell proteins, providing a measure for accumulated oxidative damage inside cells, was similar or even slightly more augmented by kaolin than by bentonite (on a mass per volume basis, Bahl et al., manuscript submitted). To put these differences into perspective, we recall that the two nanoforms of kaolin differed much less, with very similar dissolution kinetics (9.8 and 7.8 ng/cm²/h respectively, Keller et al., this Special Issue), similar oxidative damage (13 and 16 nmol TEU/m² respectively), and similar NR8383 reactivity. Thus, by the same criteria that identify the two nanoforms of kaolin as to be similar, bentonite is clearly dissimilar. Overall, bentonite proved to be the more reactive nanomaterial in many assays and, therefore, has a high capacity to cause protein damage. Indeed, we obtained first evidence for an increase in nitrosylated proteins in bentonite-treated lungs (Figure S3). Further studies should investigate if nitrosylated sites are co-localized with bentonite particles in the tissue. 

## 5. Conclusions

The paper shows that, despite highly similar composition, plate-like structure, and adsorption properties, nano-sized bentonite is far more bioactive than size-matched kaolin, and this finding strongly argues against a common grouping of both aluminosilicates. Combining the findings from the literature and also from recent studies on reactivity and solubility (see Keller et al., this Special Issue), this difference may be due to differences in ion exchange capacity, oxidative potential, specific surface area, and dissolution, as all these properties were more pronounced for bentonite compared to kaolin. The unexpectedly fast recovery found for bentonite-treated lungs may rely on a transition of bentonite from a cytotoxic to a cell-compatible material. Together, transformation and dissolution processes of aluminosilicates are highly relevant for the field of nanotoxicology and deserve further attention.

## Figures and Tables

**Table 1:** Hydrodynamic particle diameter and z-potential of kaolin and bentonite.

Value	Bentonite	Kaolin	Bentonite	Kaolin	Bentonite	Kaolin
	in H_2_O		in F-12K Medium		in KRPG	
**Mode [nm]**	141.0 ± 13.5	165.8 ± 3.9	350.9 ± 51.8	208.9 ± 54^1)^	218.5 ± 28.5	200.4 ± 65.4
**d10 [nm]**	102.8 ± 9.3	131.9 ± 0.9	201.6 ± 9.6	124.9 ± 13.7^1)^	174.1 ± 6.4	111 ± 14.9
**d50 [nm]**	139.8 ± 8.0	185.3 ± 2.7	318.3 ± 12.1	234.2 ± 13.2^1)^	262.7 ± 11.2	229.2 ± 14.2
**d90 [nm]**	190.7 ± 8.8	294.8 ± 1.6	447.9 ± 20.9	327.4 ± 6.0^1)^	401.6 ± 9.4	360.5 ± 5.3
**z-Potential [mV]**	−47.4	−30.0	−18.2	−16.7	−31.8	−31.5

^1)^ Values measured after additional brief ultra-sonication in F-12K medium. Values for d10, d50, and d90 describe the cumulative particle size distribution at 10%, 50%, and 90% of the maximum value. Particle sizes represent mean values ± standard error (n = 3); z-potential measurements represent single measurements.

**Table 2:** In vitro effects of corundum, quartz DQ12, kaolin, and bentonite on NR8383 macrophages.

Material		LDH [% of CTR]^1)^	GLU [% of CTR]^1)^	H_2_O_2_ [µmol/L]	TNFα [pg/mL] or [% cell eath]^2)^
	µg/mL	mean ± SD	mean ± SD	mean ± SD	mean ± SD
**Corundum**	0	26.1 ± 3.6	3.3 ± 0.6	0.5 ± 0.1	7.8 ± 2.3
	22.5	24.0 ± 0.6	1.1 ± 1.3	0.2 ± 0.1	8.5 ± 8.5
	45	27.1 ± 0.6	1.5 ± 1.4	0.7 ± 0.1	10.9 ± 10.8
	90	28.2 ± 2.2	1.4 ± 1.5	1.8 ± 0.1***	13.0 ± 12.1
	180	32.3 ± 0.7**	2.0 ± 1.9	3.4 ± 0.2***	21.4 ± 25.3
**Quartz**	0	26.1 ± 3.6	3.3 ± 0.6	0.5 ± 0.1	7.8 ± 2.3
**DQ12**	22.5	29.9 ± 0.6	3.5 ± 0.3	0.0 ± 0.0	13.3 ± 6.6
	45	46.7 ± 0.9***	6.6 ± 0.2**	0.2 ± 0.2	31.8 ± 3.8
	90	82.4 ± 0.3***	22.1 ± 0.2***	0.6 ± 0.1	100.7 ± 20.3**
	180	103.2 ± 0.2***	44.1 ± 0.5***	1.2 ± 0.3***	104.6 ± 47.1**
**Kaolin**	0	26.1 ± 3.6	3.3 ± 0.6	0.5 ± 0.1	7.8 ± 2.3
	2.8	38.0 ± 6.0	4.6 ± 0.4	0.2 ± 0.1	
	5.625	34.5 ± 9.3	4.4 ± 1.6	0.3 ± 0.1	
	11.25	31.9 ± 3.2	4.1 ± 0.6	0.5 ± 0.1	
	22.5	29.0 ± 3.9	4.4 ± 0.4	0.5 ± 0.2	5.6 ± 6.9
	45	51.0 ± 8.9***	8.8 ± 1.7***	1.1 ± 0.1**	19.5 ± 13.7
	90	79.6 ± 2.4***	21.9 ± 0.7***	1.9 ± 0.2***	158.3 ± 101.6***
	180	93.6 ± 3.5***	32.4 ± 2.0***	2.1 ± 0.3***	142.4 ± 81.9***
**Bentonite**	0	26.1 ± 3.6	3.3 ± 0.6	0.5 ± 0.1	19.1 ± 10.2
	2.8	20.0 ± 2.8	4.2 ± 2.5	0.6 ± 0.1	
	5.625	21.7 ± 0.8	3.8 ± 1.6	1.3 ± 0.1***	
	11.25	41.0 ± 3.1*	5.0 ± 0.7	1.7 ± 0.2***	
	22.5	70.3 ± 3.1***	13.9 ± 0.8***	2.0 ± 0.5***	70.3 ± 16.4
	45	104.7 ± 6.7***	36.2 ± 2.2***	1.9 ± 0.1***	99.1 ± 2.2*
	90	97.3 ± 3.9***	33.2 ± 1.6***	1.5 ± 0.1***	100.1 ± 2.1*
	180	43.6 ± 1.9**	28.7 ± 1.0***	1.6 ± 0.1***	100.8 ± 1.4*

^1)^ Lactate dehydrogenase activity (LDH) and glucuronidase activity (GLU) in % of triton X-100 lysed positive control values (CTR). ^2)^ TNFα values of bentonite treatment represent % cell death of L-929 reporter cells. *: *p* < 0.05; **: *p* < 0.01; ***: *p* < 0.001.

## Supplementary Materials

The following are available online at https://www.mdpi.com/2079-4991/10/2/204/s1, Table S1: Differential cell counts in bronchoalveolar lavage fluid [x 106]. Table S2: Total protein in bronchoalveolar lavage fluid. Table S3: Fibronectin in bronchoalveolar lavage fluid. Figure S1: Histologic and dark field aspects of rat lungs treated with kaolin, bentonite or quartz DQ12. Figure S2: Macroscopic inspection of bentonite-treated lungs. Figure S3: Detection of nitrosylated proteins in the bentonite-treated and control lung tissue.
