# Supplementary material for: Lung Toxicity Analysis of Nano-Sized Kaolin and Bentonite: Missing Indications for a Common Grouping
Source: Nanomaterials (Basel). 2020 Jan 24;10(2):204. doi: 10.3390/nano10020204 (PMC7075023; doi:10.3390/nano10020204)
Supplement: Supplementary file 1 [file nanomaterials-10-00204-s001.pdf]

Supplementary Material to

Lung Toxicity Analysis of Nano-Sized Kaolin and Bentonite: Missing Indications for a Common Grouping

Martin Wiemann [1*], Antje Vennemann [1] and Wendel Wohlleben[2]

[1] IBE R&D Institute for Lung Health gGmbH, Münster, Germany; martin. wiemann@ibe-ms.de; vennemann@ibe-ms.de

[2] BASF SE, Advanced Materials Research, 67056 Ludwigshafen, Germany; wendel.wohlleben@basf.com

* IBE R&D Institute for Lung Health gGmbH, Münster, Germany; martin. wiemann@ibe-ms.de

Table S1. Differential cell counts in broncho-alveolar lavage fluid [x 10^6]

	3 days						21 days					
Particle	AM		PMN		EO		AM		PMN		EO	
	mean	SD	mean	SD	mean	SD	mean	SD	mean	SD	mean	SD
**CTR**	2.23	1.08	0.00	0.00	0.06	0.06	4.64	0.49	0.00	0.00	0.00	0.00
**Quartz DQ12**	9.72	0.00	1.41	0.00	0.91	0.00	15.10	4.47	5.00	1.49	0.00	0.00
**Kaolin**	10.19	4.64	0.01	0.02	0.90	0.64	6.20	0.87	0.00	0.00	0.00	0.00
**Bentonite**	12.44	5.55	4.41	1.64	0.71	0.35	10.92	1.99	0.00	0.00	0.00	0.00

Footnotes to Table S1. CTR: vehicle control. AM, PMN, EO: counts of alveolar macrophages, polymorphonuclear granulocytes, and eosinophilic granulocytes in the broncho-alveolar lavage fluid. Mean values and standard deviation (SD) from n=5 animals.

Table S2. Total protein in broncho-alveolar lavage fluid

	3 days		21 days	
Particle	Protein [µg/mL]		Protein [µg/mL]	
	mean	SD	mean	SD
**CTR**	209.60	20.81	142.60	3.17
**Quartz DQ12**	361.20	74.22	318.50	34.25
**Kaolin**	253.20	74.61	159.40	22.45
**Bentonite**	583.70	209.60	180.20	21.28

Footnotes to Table S2. CTR: vehicle control. Mean values and standard deviation (SD) from n=5 animals.

Table S3. Fibronectin in broncho-alveolar lavage fluid

	3 days		21 days	
	Fibronectin [ng/mL]		Fibronectin [ng/mL]	
Particle	mean	SD	mean	SD
**CTR**	63.23	27.09	66.33	56.28
**Quartz DQ12**	281.00	115.60	242.10	45.55
**Kaolin**	270.30	189.70	44.78	40.08
**Bentonite**	491.20	76.66	61.02	23.66

Footnotes to Table S3. CTR: vehicle control. Mean values and standard deviation (SD) from n=5 animals.

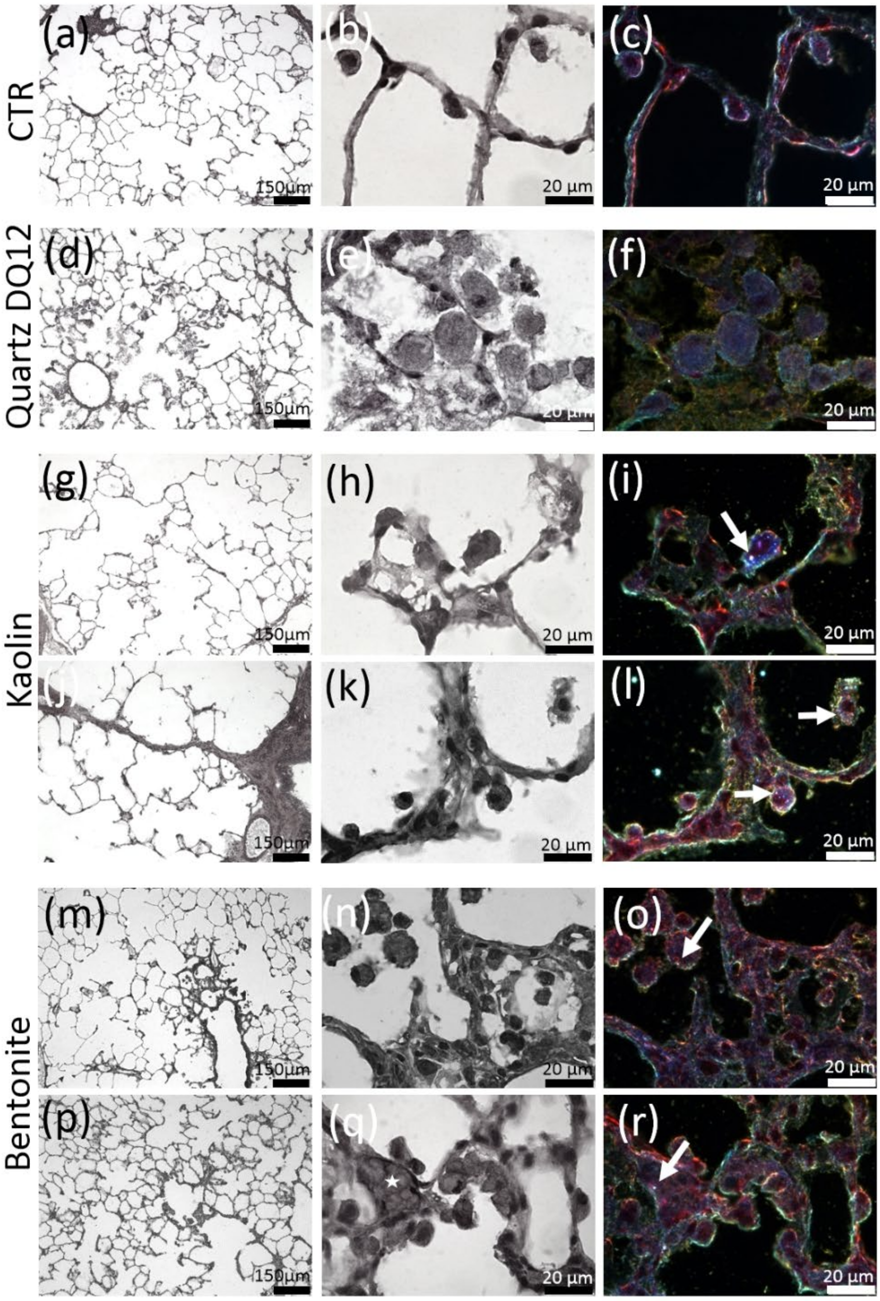

Figure S1. Histologic and dark field aspects of rat lungs treated with kaolin, bentonite or quartz DQ12. Animals were intratracheally instilled with vehicle (a-c, CTR), or 1.2 mg of either quartz DQ12 (d-f), kaolin (g-l), or bentonite (m-r) and sacrificed after 3 d (a-c, g-i, m-o) and 21 days (d-f, j-l, p-r). Arrows in i, l, o, and r point to light scattering inclusions visualized with enhanced darkfield microscopy (c, f, i, l, o, r) after administration of kaolin and bentonite. Hypercellular regions with numerous macrophages are found in quartz-treated (d-f) and bentonite-treated lungs (m-r). The latter treatment also led to intra-septal bubble-like inclusion (q, white asterisk).

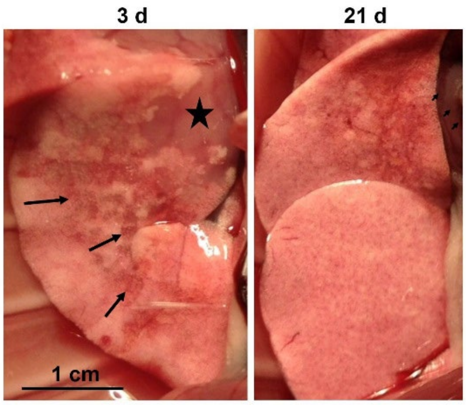

Figure S2. Macroscopic inspection of bentonite-treated lungs. Small (arrows) and confluent opaque areas (asterisk) are visible on the lung surface on day 3 (left) but no longer on day 21 (right).

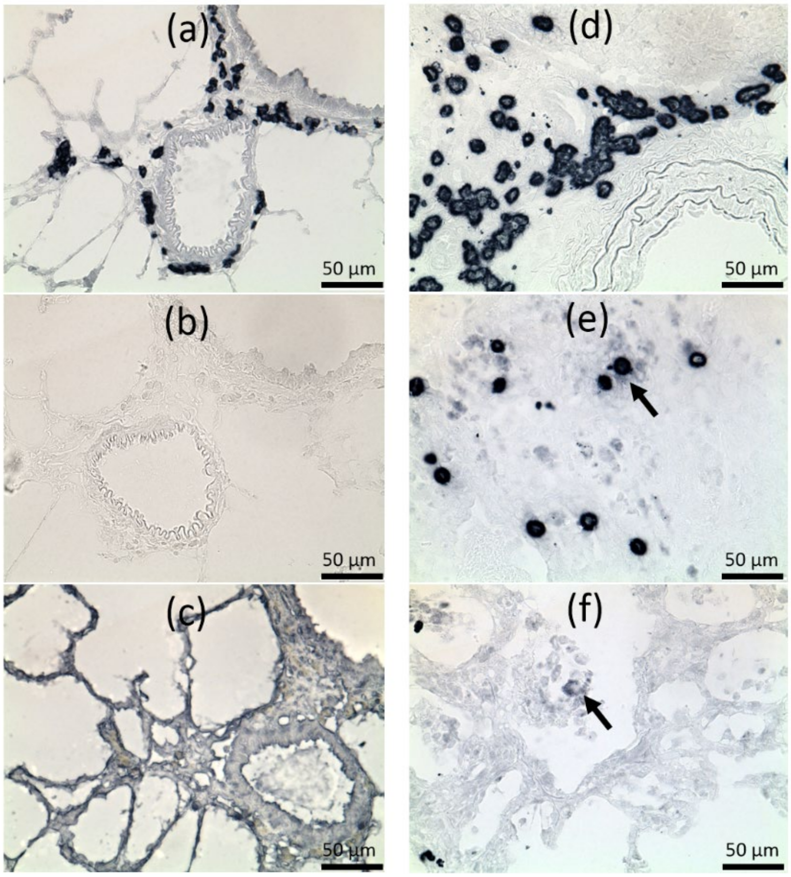

Figure S3. Detection of nitrosylated proteins in the bentonite-treated and control lung tissue. Immunocytochemical staining of cryo-sections stained with a specific anti-nitrotyrosine antibody (Merck Millipore 06-284), anti-rabbit peroxidase coupled secondary antibody und Vector Blue peroxidase stain. (a-c) particle-free control lung tissue. (a) Granulocytes within perivascular space stain heavily. (b) Control without the first specific antibody. (c) Positive control after nitrosylation reaction of the tissue (incubation in 1 mM $NaNO_2$, 100 mM Natriumacetat, and 1 mM H_2O_2, pH5); note the strong immunoreaction of all elements of the lung parenchyma. (d-f) bentonite-treated lung (1.2 mg, day 3) (d) Perivascular granulocytes stain heavily, as in controls. (f) In addition, immunopositive material occurs in the vicinity of granulocytes, and (f, arrow) in macrophage-like cell assemblies (e, arrows).
